# Nanog1 in NTERA-2 and Recombinant NanogP8 from Somatic Cancer Cells Adopt Multiple Protein Conformations and Migrate at Multiple M.W Species

**DOI:** 10.1371/journal.pone.0090615

**Published:** 2014-03-05

**Authors:** Bigang Liu, Mark D. Badeaux, Grace Choy, Dhyan Chandra, Irvin Shen, Collene R. Jeter, Kiera Rycaj, Chia-Fang Lee, Maria D. Person, Can Liu, Yueping Chen, Jianjun Shen, Sung Yun Jung, Jun Qin, Dean G. Tang

**Affiliations:** 1 Department of Molecular Carcinogenesis, University of Texas M.D Anderson Cancer Center, Science Park, Smithville, Texas, United States of America; 2 College of Pharmacy, University of Texas, Austin, Texas, United States of America; 3 Department of Biochemistry, Baylor College of Medicine, Houston, Texas, United States of America; 4 Cancer Stem Cell Institute, Research Center for Translational Medicine, East Hospital, Tongji University School of Medicine, Shanghai, China; UCSF / VA Medical Center, United States of America

## Abstract

Human Nanog1 is a 305-amino acid (aa) homeodomain-containing transcription factor critical for the pluripotency of embryonic stem (ES) and embryonal carcinoma (EC) cells. Somatic cancer cells predominantly express a retrogene homolog of Nanog1 called NanogP8, which is ∼99% similar to Nanog at the aa level. Although the predicted M.W of Nanog1/NanogP8 is ∼35 kD, both have been reported to migrate, on Western blotting (WB), at apparent molecular masses of 29–80 kD. Whether all these reported protein bands represent authentic Nanog proteins is unclear. Furthermore, detailed biochemical studies on Nanog1/NanogpP8 have been lacking. By combining WB using 8 anti-Nanog1 antibodies, immunoprecipitation, mass spectrometry, and studies using recombinant proteins, here we provide *direct* evidence that the Nanog1 protein in NTERA-2 EC cells exists as multiple M.W species from ∼22 kD to 100 kD with a major 42 kD band detectable on WB. We then demonstrate that recombinant NanogP8 (rNanogP8) proteins made in bacteria using cDNAs from multiple cancer cells also migrate, on denaturing SDS-PAGE, at ∼28 kD to 180 kD. Interestingly, different anti-Nanog1 antibodies exhibit differential reactivity towards rNanogP8 proteins, which can spontaneously form high M.W protein species. Finally, we show that most long-term cultured cancer cell lines seem to express very low levels of or different endogenous NanogP8 protein that cannot be readily detected by immunoprecipitation. Altogether, the current study reveals unique biochemical properties of Nanog1 in EC cells and NanogP8 in somatic cancer cells.

## Introduction

Nanog1 (commonly called Nanog) is encoded by the *nanog* gene located on Chr. 12p13.31 (Fig. S1A). The gene has 4 exons and encodes a homeodomain transcription factor that is crucial for the self-renewal of embryonic stem (ES) cells [1], [2]. Nanog1 overexpression in mouse ES cells (mESCs) overcomes the requirement of leukemia inhibitory factor for maintaining the pluripotency [1], [3] whereas disruption of *nanog* results in mESC differentiation to extraembryonic endoderm [4]. Down-regulation of Nanog1 in human ESCs (hESCs) also leads to the loss of pluripotency and differentiation to extraembryonic cell lineages [5]. Furthermore, in association with other reprogramming factors, Nanog1 overcomes reprogramming barriers and promotes somatic cell reprogramming [6], [7]. Thus, Nanog1 is a core intrinsic element of the transcriptional network for sustaining the self-renewal of ESCs.

**Figure 1:**
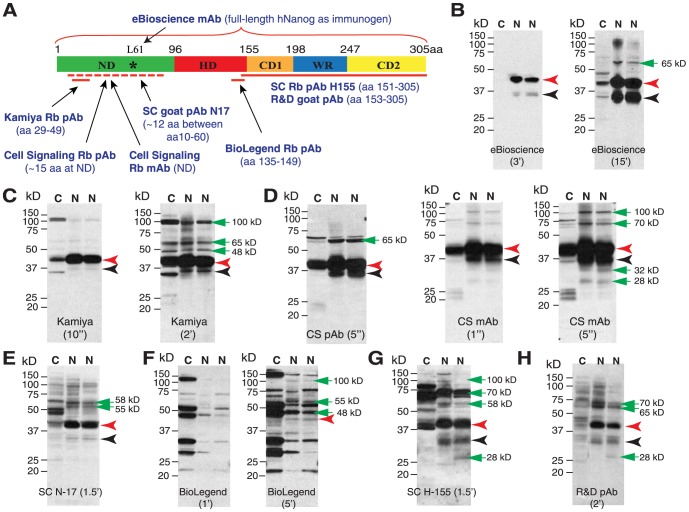
WB analysis of endogenous Nanog1 protein species in NTERA-2 cells. (**A**) Schematic of the human Nanog protein and 8 anti-Nanog Abs used in this study. Shown in parentheses are epitopes of individual Abs. ND, N-terminus domain; HD, homeodomain; CD1 and CD2, C-terminus domain 1 and 2; WR, tryptophan-rich domain. The asterisk in ND indicates the Leu61 residue recognized by the eBioscience mAb (arrow) mapped from our present studies. (B–H) WB analysis in NTERA-2 NE (N, two different batches) or cytosol (C) using 8 anti-Nanog Abs. Individual Ab is indicated at the bottom and M.W on the left. Black arrowhead, the predicted 35 kD Nanog protein; red arrowhead, the main 42 kD band; green arrows, additional bands (especially after longer exposures).

Human Nanog1 protein has 305 amino acids (aa) and 5 functional subdomains, i.e., N-terminal domain (ND), homeodomain (HD), C1-terminal domain (CD1), tryptophan-rich domain (WR) and C2-terminal domain (CD2) [8]–[11] (Fig. 1A). The ND is involved in transcription interference and C-terminal region contains the transcription activator. The HD domain is required for Nanog nuclear localization and transactivation and the WR region mediates the dimerization of Nanog protein, which is required for pluripotency activity [12], [13]. Of interest, human *Nanog* has 11 pseudogenes [14], among which *NanogP8*, located on Chr. 15q14, has a complete open reading frame [14], [15] (Fig. S1B) that possesses an Alu element in the 3′-UTR homologous to the one in *Nanog1* gene, suggesting that *NanogP8* is a retrogene rather than a pseudogene. NanogP8 has at least 6 conserved nucleotide (nt) differences compared to Nanog1, which may result in some aa changes [15].

Interestingly, many somatic cancer cells have been reported to express Nanog mRNA and/or protein [15]–[46]. Several caveats are associated with many of these studies. ***First***, at the mRNA level, rigorous studies employing differential RT-PCR combined with sequencing or differential sensitivity to the restriction enzyme AlwN1 [15], [17], [31], [32], [34], [46], have demonstrated that somatic cancer cells preferentially express the transcript of the *NanogP8* gene (Fig. S1B; see below) rather than *Nanog1*. Indeed, we have observed that the *nanog1* locus is silenced in some somatic cancer cells [15]. Our sequencing analyses reveal that embryonal carcinoma (EC) NTERA-2 cells express *Nanog1* but not *NanogP8* mRNA whereas all somatic cancer cells (6 different types including primary prostate tumor-derived cells) show 5 of the 6 nt differences specific to *NanogP8* [15]. Among the 5 conserved nt changes in *NanogP8*, one (nt759) should result in aa change (Q253H) although some cancer cells show other non-conserved nt changes (i.e., polymorphisms) that could also result in aa changes (e.g., L61P for HPCa6) [15]. Making the distinction between Nanog1 and NanogP8 is important because the two transcripts are derived from separate genomic loci and have differences at the nt sequence levels.

***Second***, many previous studies have been merely correlative without probing the functional importance of NanogP8 expression in cancer cells. Using human prostate cancer (PCa) as a model, we have shown [15] that: 1) NanogP8 protein is expressed as a gradient in PCa cells with readily detectable nuclear NanogP8 in only a small fraction of PCa cells; 2) NanogP8 protein-expressing cells are increased in primary PCa compared to matching benign tissues; 3) *NanogP8* mRNA and NanogP8 protein are enriched in CD44^+^ and CD44^+^CD133^+^ primary PCa cells; 4) shRNA-mediated knockdown of *NanogP8* inhibits tumor regeneration of prostate, breast, and colon cancer cells; and 5) The tumor-inhibitory effects of *NanogP8* knockdown are associated with inhibition of cell proliferation and clonal expansion of tumor cells and disruption of differentiation. Our recent studies demonstrate that inducible NanogP8 expression in bulk PCa cells is sufficient to confer cancer stem cell (CSC) properties and promote androgen-independent PCa growth [34] and that NanogP8 is enriched in undifferentiated (PSA^-/lo^) PCa cells and its knockdown retards outgrowth of castration-resistant PCa [41]. Our studies [15], [34], [41] point to potential pro-oncogenic functions of NanogP8.

***Third***, the predicted Nanog1 and NanogP8 proteins are ∼99% identical [15]. Consequently, the term ‘Nanog’ is often used to generically refer to either protein (that is also the case in this paper). Nevertheless, the specificity of the majority of commercially available anti-Nanog antibodies (which were all raised against Nanog1; Table 1) for Nanog1 and in particular, for NanogP8 remains uncharacterized. Therefore, it is unclear whether the putative Nanog protein bands shown on Western blotting (WB), in which frequently only a cropped strip is shown, or the Nanog protein staining shown in immunohistochemistry (IHC) truly represent the Nanog protein. ***Finally***, intriguingly, although the predicated molecular mass of Nanog protein (for both Nanog1 and NanogP8) is ∼35 kD, numerous studies have reported putative Nanog proteins migrating, on SDS-PAGE, at apparent molecular mass of 29 to 80 kD in ES, EC, and somatic cancer cells (Table 1)[2], [5], [17], [24]–[26], [46]–[59]. Even more puzzlingly, the same antibody often seems to detect different ‘Nanog’ protein bands (Table 1). Whether all these reported putative Nanog protein species are true Nanog proteins has yet to be *directly* determined.

**Table 1:** Examples of Nanog antibodies and their recognized protein bands.

Antibody*	Remarks	Protein band(s)	Reference(s)
**eBioscience mAb** (Cat# 14-5768)	Affinity-purified mAb using full-length hNanog as immunogen	∼**43 kD** band in N-tera lysate; 2 **bands** of unspecified M.W	Product sheet, 24
**Kamiya Rb pAb** (PC-102)	Affinity-purified pAb using hNanog aa 29-49 as immunogen	∼**42 kD band** in hESCs	Product sheet
**SC goat pAb-N17** (SC-30331; N17)	Affinity-purified pAb against a hNanog N-terminus peptide	∼**43 kD band** using rhNanog;∼**34 kD** in cancer cell lysates	Product sheet, 25
**Cell Signaling pAb** (3580)	Affinity-purified Rb pAb against N-terminus of hNanog	**42∼45 kD** band in N-tera lysate	Product sheet
**Cell Signaling mAb** (5232)	Affinity-purified Rb mAb against N-terminus of hNanog	This antibody is used for ChIP	Product sheet
**BioLegend Rb pAb** (632002)	Affinity-purified pAb against hNanog aa 135-149	∼**46 kD band** in N-tera cells	Product sheet
**SC Rb pAb-H155** (SC-33759; H155)	Using hNanog aa151-305 peptide as immunogen	**40 kD band** in human ES cells; ∼**40 kD** in adipose-derived stem cells; ∼**40 kD** in lymphoma cell lines.	Product sheet, 47, 33
**R & D goat pAb** (AF1997)	Affinity-purified pAb against rhNanog aa 153-305 peptide	**40∼42 kD** band using rhNanog; **35 kD** band in hESCs & EC cells; Several bands at ≥**34 kD** in HepG2 & OS732 cells; 36∼**37 kD** in trophoblastic samples; ∼**42 kD** in human colorectal cancer	Product sheet, 5, 16, 48, 26, 46
**Abcam Rb pAb** (21624)	Same as Kamiya pAb	∼**40 kD** band in hESCs; A cluster of bands (unknown MW)	Product sheet, 49
**Abcam Rb pAb** (21603)	Using full-length mNanog as immunogen	∼**39 kD** band in mESCs A cluster of bands (unknown MW) ∼**38 kD** band in hESCs	Product sheet, 50, 51
**Abnova mouse mAb** (Clone 2C11)	Raised against C-terminal Nanog	**48 kD**, **35 kD** and **29 kD** bands in N-tera cells	52
**Bethyl Labs Rb pAb** (BL1662)	Affinity purified pAb raised against mNanog	At least two bands between **37** and **49 kD** in mESCs	53
**Chemicon Rb pAb** (AB5731)	Affinity purified pAb raised against mNanog N-terminus peptide	∼**35 and 55 kD** bands in mESCs; A cluster of bands in mESCs	Product sheet, 54
**Chemicon Rb pAb**	Raised against mNanog	**40 kD** and **80 kD** bands in mESCs; Dog testis and pig testis	55
**Home-made Rb pAb**	Raised against mNanog	2-3 bands at ∼**40 kD** in mESCs	2
**Home-made Rb pAb**	Raised against mNanog	>3 bands with the largest at ∼**44 kD** in mESCs, and EG cells	56
**Home-made Rb pAb**	Raised against hNanog (aa 168-183)	>3 bands of ∼**36 kD** to ∼**50 kD** in 293T cells transfected with hNanog	57
**Home-made Rb pAb**	FLAG-tagged Nanog stably expressed in mESCs	Two pulldown Nanog protein bands at ∼**45 kD** and **37 kD**	58
**Home-made Rb pAb**	Raised against mNanog aa 1-95	Several bands at >**35 kD**	59

*This table lists the information for the 8 antibodies used in our current study, together with several others commercial and home-made antibodies. Shown in parenthesis are catalog numbers.

**Abbreviations**: EC, embryonal carcinoma; EG, embryonic germ cells; hESCs, human embryonic stem cells; hNanog,

human Nanog; mESCs, mouse embryonic stem cells; mAb, monoclonal antibody; mNanog, mouse Nanog protein;

pAb, polyclonal antibody; Rb, rabbit; rhNanog, recombinant human Nanog protein; SC, Santa Cruz.

The present study was undertaken to address the last two questions. We first provide direct evidence that the Nanog1 protein in NTERA-2 EC cells migrates at <30 to ∼100 kD. We then show that recombinant NanogP8 proteins can also migrate at ∼28 kD to ∼180 kD. These results suggest that the Nanog1/NanogP8 proteins likely adopt multiple conformations. We finally demonstrate that most long-term cultured somatic cancer cells seem to express very low levels of or biochemically divergent endogenous NanogP8 such that it cannot be readily immunoprecipitated down by the 8 commercial anti-NanogP8 antibodies.

## Materials and Methods

### Cells and Reagents

Various human cancer cell lines, including prostate cancer (PC3, Du145, LNCaP), breast cancer (MCF-7), colonic carcinoma (Colo320), and melanoma (WM-562) cells, were obtained from ATCC (American Type Culture Collection, Manassas, VA) and cultured in the recommended media containing 10% of heat-inactivated FBS (fetal bovine serum). Teratocarcinoma cells (NTERA-2, clone D; CRL-1973) were purchased from ATCC and cultured in mTeSR™1 medium (STEMCELL Technologies, Vancouver, Canada). Nuclear extraction kit was from Thermo Scientific (Rockford, IL). Eight anti-Nanog primary antibodies (Abs) were obtained from commercial companies (Table 1; Fig. 1A). Secondary and control Abs were purchased from Santa Cruz Biotechnology. ECL reagents were bought from PerkinElmer, Inc (MA, USA). The current research does not involve animal experiments or human subjects (i.e., living individuals or identifiable private information). All other studies presented herein were the investigator-initiated and did not require approval from other regulatory bodies.

### siRNA- and shRNA-mediated Nanog knockdown experiments

**Figure 2:**
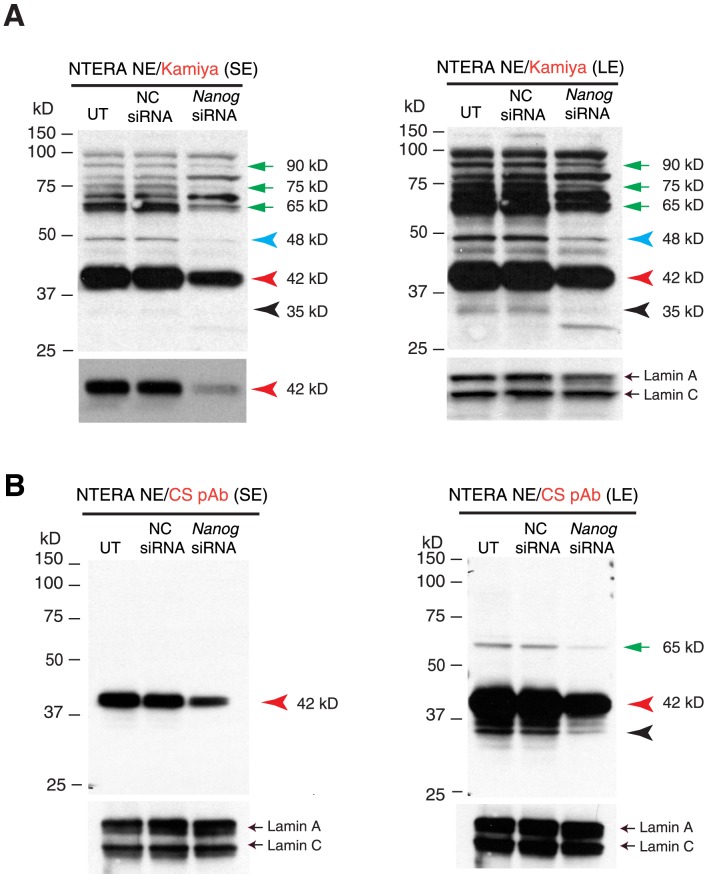
Characterization of Nanog proteins in NTERA-2 cells upon siRNA-mediated knockdown. (**A**) Nanog siRNA experiments in NTERA-2 cells. NTERA-2 cells were transfected with a pool of Nanog-specific siRNAs for 48 h, harvested, and used to isolate the NE for WB with the Kamiya Ab. The left and right panels represent the short and long exposure (SE and LE, respectively) films (note that the left bottom panel is the shortest exposed film to illustrate the significant knockdown of the 42 kD band). Lamin A/C was the loading control. UT, untransfected; NC siRNA, negative control (siCONTROL non-targeting) siRNAs; Nanog siRNA, SMARTpool siRNA against Nanog. Red, black, and blue arrowheads indicate the major 42 kD, minor 35 kD, and 48 kD protein bands, respectively, that were reduced upon Nanog knockdown. The green arrows refer to additional bands that also showed reduction. (**B**) The WB was performed with the CS anti-Nanog rabbit pAb.

For the siRNA knockdown experiments (Fig. 2), we used siGENOME SMARTpool siRNA against human Nanog1 and siCONTROL non-targeting siRNAs obtained from Dharmacon (Lafayette, CO). Cells plated 24 h earlier on 6-well dishes were transfected with siRNAs using Lipofectamine 2000. 48 h later, cells were harvested for WB experiments.

For the shRNA Nanog knockdown experiments, lentiviruses including pLL3.7, Nanog-shRNA and TRC-shRNA were produced in 293FT packaging cells (Clontech Laboratories, Mountain View, CA) using the modified protocols previously described [15], [60]. In brief, geneticin-selected, early-passage 293FT cells (6×10^6^/15 cm dish) were transfected with the RRE (6 µg), REV (4 µg) and VSVg (4 µg) packaging plasmids, along with a lentiviral vector (6 µg) using Lipofectamine 2000 to carry out the transfection. At 36–48 h, virus-containing media were collected and fresh media added. After an additional 12–24 h of culture, viral supernatants were again collected, pooled, and ultracentrifuged to produce concentrated viral stocks. Individual titers were determined for the GFP-tagged virus using HT1080 cells. The non-GFP tagged TRC-shRNA virus was prepared in parallel and assigned the control (pLL3.7) titer. Target cells were plated 24 h earlier and infected at approximately 50% cell density and harvested for *in vitro* or *in vivo* experiments 48–72 h post infection.

### Bacterial expression and purification of recombinant Nanog (rNanog) proteins

*NanogP8* or *Nanog1* cDNA in cultured NTERA-2, MCF-7, and LNCaP cells or in primary human PCa samples (HPCa1, HPCa5, and HPCa6) was amplified by RT-PCR using LDF1/LDR1 primers (LDF1 5′-TCTTCCTCTATACTAACATGAGT-3′; LDR1 5′-AGGATTCAGCCAGTGTCCA-3′) and cloned into pCR2.1 [15]. The EcoRI/NotI or BamHI/SalI fragments containing the Nanog coding sequence were then subcloned into the same sites in either pET-28a (+) or pET-28b (+) bacterial expression vector to generate His-tagged fusion proteins. After insert verification by restriction enzyme digestion analysis and DNA sequencing, the plasmids were used to transform BL21(DE)_3_ competent bacteria to express the fusion proteins. Nanog protein was induced by 0.5 mM IPTG for 3–6 h at 37°C and bacterial pellet was kept at −80°C. For purification, bacterial pellets containing His-tagged Nanog protein were lysed in lysis buffer (50 mM NaH_2_P0_4_, pH 8.0, 300 mM NaCl, 10 mM imidazole, protease inhibitor cocktail, and 1 mg/ml lysozyme), sonicated for 10 sec (one pulse). His-tagged Nanog in the supernatant was purified by nickel beads per manufacturer's instructions (Qiagen).

### WB using recombinant proteins (rNanog), whole cell lysate (WCL), or nuclear extract (NE)

rNanog proteins were obtained and purified as described above. WCL and NE (from cultured cells) were prepared as previously described [15]. 50–80 µg proteins (rNanog and NTERA-2 NE) were analyzed by 12.5% SDS-PAGE and the gels were transferred onto an immobilon-P transfer membrane (Millipore, Bedford, MA). The membrane was blocked with 5% non-fat dried milk in TBST (10 mM Tris-HCl, 150 mM NaCl and 0.1% Tween-20) for 1 h at room temperature, and incubated overnight at 4°C with primary antibody. Membranes were washed three times with TBST buffer, then incubated for 1 h with 1∶2000 secondary antibody, and developed with ECL Plus WB detection reagent (PerkinElmer).

### Immunoprecipitation (IP)

Recombinant proteins (500 µg) or NE (800 µg or depending on experimental purposes) were incubated with the indicated anti-Nanog antibodies overnight at 4°C. Then the solution was incubated with protein A/G-agarose (Sigma, MO, USA) for another 1 h at 4°C. After incubation, the beads were washed three times with RIPA buffer. Proteins bound to protein A-agarose were eluted with SDS-PAGE loading buffer and boiled for 8 min and subjected to SDS-PAGE.

### Dialysis refolding experiments

To isolate and purify a large amount of rNanog, bacterial pellets were sonicated 6 times with 10 seconds pulses. The sonication solution was centrifuged at 10,000 rpm for 15 min at 4°C. The pellets were washed with cold PBS for three times. The precipitated material was the inclusion body of rNanog protein. To dissolve the inclusion body, a buffer containing 7 M urea was used and then the solution was subjected to dialysis against the buffers of 1.5 M, 0.6 M, 0.3 M, and 0.1 M urea (in 1.5 M NaCl, 10 mM Tris HCl, pH 7.0). The dialysis solution was subjected to SDS-PAGE to determine the existence of soluble Nanog protein.

### Nanog protein identification (ID) by mass spectrometry (MS) analyses

Three sets of MS-based protein ID experiments were performed on either rNanog proteins or NE prepared from NTERA-2 cells. In the ***first*** ID experiment, we immunoprecipitated a large amount of NTERA-2 cell NE (∼2 mg total) with the R&D goat pAb. After washing (3x) with RIPA buffer, the proteins bound to the protein G-agarose were eluted with SDS-PAGE loading buffer and boiled for 8 min and subjected to SDS-PAGE. The gel was then stained with SYPRO Ruby solution. Gel slices/areas containing bands of interest were cut out, proteins eluted, and subjected to trypsin digestion. The tryptic digests were analyzed by LC-MS/MS using the Dionex Ultimate 3000 RSLCnano LC coupled to the Thermo Orbitrap Elite. Prior to HPLC separation, the peptides were desalted using Millipore U-C18 ZipTip Pipette Tips following the manufacturer's protocol. A 2 cm long×100 µm I.D. C18 5 µm trap column (Proxeon EASY Column) was followed by a 75 µm I.D. ×15 cm long analytical column packed with C18 3 µm material (Dionex Acclaim PepMap 100). Buffer A was composed of 0.1% formic acid in water and Buffer B 0.1% formic acid in acetonitrile. Data was acquired for 35 min using an HPLC gradient of 5% B to 45% B over 30 min with a flow rate of 300 nl/min. The FT-MS resolution is set to 120,000, and top 20 MS/MS are acquired in CID ion trap mode. Raw data was processed using SEQUEST embedded in Proteome Discoverer v1.3 using the following parameters: full trypsin digest with maximum 2 missed cleavages, fixed modification carbamidomethylation of cysteine, variable modification oxidation of methionine and deaminidation of asparagine and glutamine, searching the human reference proteome from Uniprot from March 2012 (80990 entries). The mass accuracy for precursors was set to 10 ppm monoisotopic mass, for fragment ions was 0.8 Da monoisotopic mass. A decoy database was generated from the Uniprot human database and used by Peptide Validator and Scaffold for calculating false positive values. X!Tandem database searches (The GPM, thegpm.org, version CYCLONE (2010.12.01.1)) were performed embedded in Scaffold 3 Q+ (Proteome Software) using the same search parameters as SEQUEST. Scaffold was used for validation of peptide and protein identifications with confidence filtering of 95% confidence for two peptides, and a 99.9% protein confidence cutoff. False positive peptide and protein values were calculated as 0.02% and 0.1% respectively by Scaffold.

In the ***second*** set of experiments using the NTERA-2 cell NE, we performed the tandem IP using the Kamiya pAb followed by the R&D pAb and the immunoprecipitates were separated on a linear ion-trap mass spectrometry (LTQ, Oorbitrap Velos) as described [61]. Simply, the excised fragments were destained with destaining solution (40% methanol, 50 mm NaHCO_3_ in water) and subjected to in-gel digestion using 100 ng trypsin in 50 mM NH_4_HCO_3_, pH 8.5, for 12 h. Peptides were then extracted with acetonitrile and dried in a Speed-Vac dryer (Thermo Savant). Each dried sample was dissolved in 20 µl of 5% methanol/95% water/0.01% formic acid solution and injected into the Surveyor HPLC system (Thermo Finnigan) using an autosampler. A 100 mm×75 µm C18 column (5 µm, 300 Å pore diameter, PicoFrit; New Objective) with mobile phases of A (0.1% formic acid in water) and B (0.1% formic acid in methanol) was used, with a gradient of 5–95% of phase B over 45 min followed by 95% of phase B for 5 min at 200 nl/min. Peptides were directly electrosprayed into the (LTQ Oorbitrap Velos (Thermo Scientific) using a nanospray source. The LTQ was operated in the data-dependent mode to acquire fragmentation spectra of the 20 strongest ions under direct control of Xcalibur software (Thermo Scientific). Obtained MS/MS spectra were searched against target-decoy Human refseq database in Proteome Discoverer 1.2 interface (Thermo Fisher) with Mascot algorithm (Mascot 2.1, Matrix Science). Variable modification of Acetylation (lysine), di-Glycine (lysine), Phosphorylation (Serine and Threonine) and Oxidation (Methionine) was allowed. Also, static modification of DeStreak (Cystein) was allowed. The precursor mass tolerance was confined within 10 ppm with fragment mass tolerance of 0.5 dalton and a maximum of two missed cleavages allowed. Assigned peptides were filtered with 5% false discover rate and subject to manual verifications.

In the ***third*** ID experiment, rNanog1/rNanogP8 proteins were purified and gel slices containing various protein bands were used in MALDI-TOF/TOF identification as described previously [62]. Tryptic digests were analyzed using a 4700 Proteomics Analyzer MALDI-TOF/TOF (AB Sciex, Foster City, CA). Samples were desalted with μC18 ZipTips (Millipore, Billerica, MA) with elution directly onto the MALDI target using the matrix α-cyano-4-hydroxycinnamic acid at 5 mg/ml in 67% acetonitrile/0.1% trifluoroacetic acid. MS and MS/MS spectra were acquired automatically using 4000 Series Explorer V 3.0 RC1. Up to 20 peaks with S/N 20 were selected for MS/MS fragmentation, excluding matrix, trypsin, and keratin peaks. Additional peak processing and database search were performed using GPS Explorer v3.5. MASCOT V2.0 or V2.2. Spectra were searched against the Swiss-Prot database including Human sequences (Sept. 1, 2009, 20495 entries). The search parameters chosen were cleavage by trypsin/P with up to 2 missed cleavages, fixed modification of carbamidomethylation of cysteine and variable modifications of protein N-terminal acetylation, methionine oxidation and pyroglutamic acid modification of peptide N-terminal glutamine residues. The database search used 50 ppm mass tolerance for monoisotopic MS masses and 0.2 Da for MS/MS, up to 100 peaks with minimum S/N 15 were selected for MS, and up to 65 fragment ions with minimum S/N 3. The search output combines the scores from MS search and the MS/MS search using a probabilistic MOWSE algorithm. The MASCOT score is defined as –10*logP, where P is the probability that the observed match is a random event. The MASCOT score 56 which corresponds to p<0.05 is chosen as the cutoff for a significant hit, and those proteins exceeding the cutoff value are reported.

## Results

### Seven of the eight anti-Nanog antibodies detect, on WB, the major 42 kD and minor 35 kD bands in NTERA-2 NE

Human *Nanog1* gene (gi 13376297), localized on Chr. 12p13.31 and primarily expressed in ES and EC cells, has a 915-bp open reading frame (Fig. S1A). It encodes a 305-aa protein that is commonly referred to as Nanog (Fig. 1A). Human Nanog1 protein is predicted to have a molecular mass of ∼35 kD. Intriguingly, putative Nanog proteins migrating at apparent molecular masses of 29–80 kD on WB have been reported in ES, EC, and somatic cancer cells (Table 1). It is unclear, however, whether these reported Nanog protein species truly represent the Nanog proteins. To address this issue and to directly determine the molecular mass(es) of endogenous Nanog protein, we first performed comprehensive WB analyses in the EC NTERA-2 cells using 8 commercial anti-Nanog (i.e., anti-Nanog1) antibodies (Abs) directed against different regions of the human Nanog protein (Fig. 1A; Table 1, the first 8 Abs). Among the 8 anti-Nanog Abs, four [Kamiya Rb pAb, SC (Santa Cruz) goat pAb N17, Cell Signaling (CS) Rb pAb and Rb mAb] are directed to the ND, two (SC Rb pAb H-155 and R&D goat pAb) to the C-terminus, and one (BioLegend Rb pAb) to the HD (homeodomain) whereas another (eBioscience mAb) is raised against the full-length human Nanog1 protein (Fig. 1A).

The WB results revealed that different antibodies displayed distinct reactivity and each antibody detected multiple bands in NTERA-2 NE (we focused on nuclear expression as Nanog is a nuclear transcription factor) with M.W ranging from ∼28 kD to ∼100 kD (Fig. 1B-H). Specifically, the results demonstrated that: **1** ) the eBioscience mAb showed the *cleanest* and most *specific* reactivity, detecting only a strong band of ∼42 kD (Fig. 1B, red arrowhead) and a weak band of ∼35 kD (Fig. 1B, black arrowhead) in the NE with no immunoreactive bands in the cytosol with 3 min exposure of the film (Fig. 1B). On the other hand, the eBioscience mAb exhibited the lowest sensitivity among the 8 antibodies. The 35 kD band and another ∼65 kD band in the NE became apparent after extended exposure time (15 min; Fig. 1B). **2** ) The Kamiya pAb showed *the second cleanest* reactivity (Fig. 1C). Upon short exposure (10 sec), it detected a strong band of 42 kD with a very faint band of 35 kD in the NE (Fig. 1C; red and black arrowheads, respectively). In the cytosol, it detected the 42 kD band and several other bands (Fig. 1C). Upon longer exposure (2 min), the Kamiya pAb also detected three upper bands at ∼48 kD, ∼65 kD and ∼90 kD in both NE and cytosol (Fig. 1C, green arrows in right panel). **3** ) Both CS Rb pAb and mAb were *the most sensitive* antibodies such that they detected the prominent 42 kD and 35 kD bands with only 1-5 sec exposure (Fig. 1D). Both antibodies also detected several additional bands (Fig. 1D, green arrows). **4** ) The SC goat pAb (N17) detected an obvious 42 kD band and a faint 35 kD band, together with two upper bands of ∼55 kD and ∼58 kD (Fig. 1E). **5** ) The Biolegend Rb pAb was the ‘dirtiest’ Ab and detected a series of strong bands in the cytosol and only faint 42 kD band in the NE (Fig. 1F, red arrow). **6** ) The SC Rb pAb (H-155) detected the obvious 42 kD band and the weaker 35 kD band (Fig. 1G, red and black arrowheads, respectively), together with several other bands at 100 kD, 70 kD, 58 kD and ∼28 kD (Fig. 1G, green arrows). **7** ) Finally, the R&D goat pAb detected an apparent band of 42 kD and a minor band of 35 kD (Fig. 1H, red and black arrowheads, respectively), together with several additional bands (Fig. 1H, green arrowheads).

In summary, this comprehensive WB analysis using 8 anti-Nanog Abs and the NTERA-2 NE has revealed that: **1** ) except for the BioLegend Ab, the other 7 anti-Nanog Abs all *commonly* recognize a major 42 kD and a minor 35 kD protein band; **2** ) Three antibodies raised against the N-terminus, i.e., Kamiya Rb pAb, CS pAb and CS mAb, give clean WB results upon short exposure of films; **3** ) with longer exposure, all antibodies detect additional protein bands ranging from ∼28 kD to >100 kD. However, different Abs detect different patterns of reactive protein bands; **4** ) With respect to sensitivity, the CS mAb and CS pAb are the most sensitive followed by the Kamiya Ab whereas the eBioscience mAb is the least sensitive; **5** ) The BioLegend anti-Nanog Ab does not detect the 42 kD as the major protein band in the NTERA-2 NE; and **6** ) Different antibodies may preferentially recognize different protein bands. For example, the Kamiya pAb, but not the CS pAb or mAb reacted with the 48 kD band.

### siRNA-mediated knock-down reveals the 42-kD protein band as the predominant Nanog protein isoform in NTERA-2 cells

Since the predicted Nanog protein is ∼35 kD, we can infer that the minor 35 kD protein band commonly detected on WB most likely is the Nanog protein. To confirm this inference and to determine whether the predominant 42 kD and any of the additional bands are also Nanog proteins, we performed siRNA knock-down experiments in NTERA-2 cells. As shown in Fig. 2A, the Kamiya Rb pAb consistently detected the strong 42 kD protein band, which decreased in response to Nanog siRNA. In addition, the minor 35 kD band and several other weaker bands migrating at 48 kD, 65 kD, 75 kD, and 90 kD all decreased in Nanog siRNA-treated cells (Fig. 2A). When we performed WB using the CS Rb pAb, the Nanog siRNAs also knocked down the 42 kD and 35 kD bands (Fig. 2B). In addition, an upper 65 kD band was also reduced (Fig. 2B). Note that the CS pAb did not detect 48 kD or other upper bands except the 65 kD band (Fig. 2B), consistent with the earlier WB results with the CS pAb and mAb (Fig. 1D). Collectively, the siRNA knock-down experiments suggest that *the Nanog protein in NTERA-2 cells seems to migrate, on denaturing SDS-PAGE, at multiple molecular masses including approximately* ***35*** *,* ***42*** *,* ***48*** *,* ***65*** *,* ***75*** *, and* ***90*** *kD with the 42-kD band being the dominant “isoform”*.

### Characterization of Nanog protein species in NTERA-2 cells by immunoprecipitation (IP) followed by mass spectrometry (MS) identification (ID)

**Figure 3:**
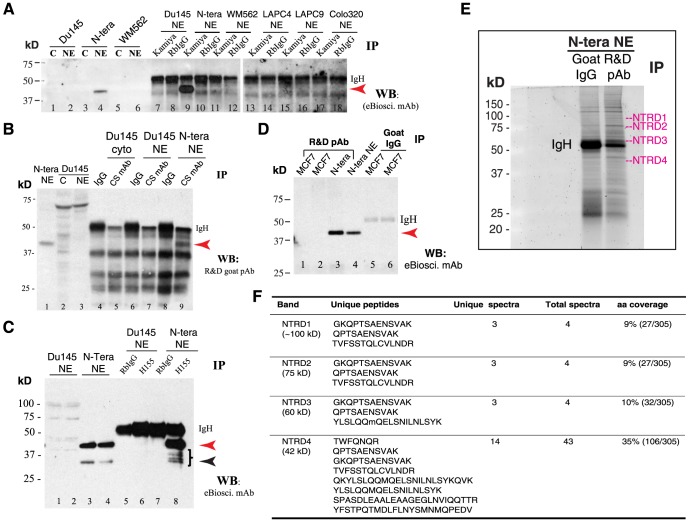
IP and ID studies with 5 anti-Nanog antibodies in NTERA-2 and cancer cells. (**A**) The NE of NTERA-2 and various cancer cells was used in IP with the Kamiya anti-Nanog Rb pAb followed by WB with the eBioscience mAb. Lanes 1-6 were regular WB with either cytosol (C) or NE. Red arrowhead, the 42 kD Nanog band; IgH, IgG heavy chain (∼53 kD). Note that the prominent 42 kD protein band was detected on WB (lane 4) and immunoprecipitated down (lane 9) *only* in NTERA-2 NE. (**B**) The NTERA-2 NE or Du145 cytosol (cyto) or NE was used in IP with the CS anti-Nanog rabbit mAb followed by WB with the R&D goat pAb. Lanes 1-3 were regular WB. Red arrowhead, the 42 kD Nanog band; IgH, IgG heavy chain. Note that the 42 kD Nanog protein was detected on WB (lane 1) and immunoprecipitated down (lane 9) only in NTERA-2 NE. (**C**) The NE of NTERA-2 and Du145 cells was used in IP with the SC anti-Nanog rabbit pAb H155 followed by WB with eBioscience mAb. Lanes 1–4 were regular WB using two independent preparations of Du145 or NTERA-2 NE. Red arrowhead, the 42-kD band; black arrowhead, the 35-kD Nanog band; IgH, IgG heavy chain. Note that the 42-kD protein band was detected on WB (lane 3 and 4) and immunoprecipitated down (lane 8) *only* in NTERA-2 NE. The right-pointing bracket indicates the cluster of Nanog protein bands below the dominant 42 kD band. (**D**) The NE of NTERA-2 cells and MCF7 cells (two independent preparations) was used in IP with the R&D anti-Nanog goat pAb (goat IgG used as the control) followed by WB with the eBioscience mAb. Red arrowhead, the 42 kD Nanog band; IgH, IgG heavy chain. Note that the 42-kD protein band was detected on WB (lane 4; input) and immunoprecipitated down (lane 3) only in NTERA-2 NE. (**E–F**) Nanog protein ID by MALDI-TOF/TOF in NTERA-2 NE following IP using the R&D goat pAb. Shown are SYPRO Ruby gel image (E; NTRD1-4 refer to the 4 gel slices cut out for protein elution) and the Nanog peptides recovered from each gel slice (F).

To substantiate the siRNA knockdown results, we performed two sets of MS-based protein ID experiments. *In the first*, we performed IP experiments using **500** µ**g** of NE from NTERA-2 and somatic cancer cells and with 5 anti-Nanog Abs, i.e., eBioscience mAb, Kamiya pAb, CS Rb mAb, SC pAb H155, and R&D goat pAb (Fig. 3; Fig. S2A-B; data not shown). All 5 antibodies immunoprecipitated the 42 kD Nanog band in NTERA-2 NE. For example, when IP was done with the Kamiya pAb followed by WB using either eBioscience mAb (Fig. 3A, lane 9) or R&D goat pAb (Fig. S2A, lane 10), the 42 kD protein was immunoprecipitated. IP with the CS Rb mAb, also directed against the N-terminus of Nanog, pulled down the 42 kD band (WB using the R&D goat pAb; Fig. 3B, lane 9). When IP was done with the SC pAb H-155, directed towards the C-terminus of Nanog, followed by WB using either eBioscience mAb (Fig. 3C, lane 8) or R&D goat pAb (Fig. S2B), the 42 kD protein was immunoprecipitated in the NTERA-2 NE. Similarly, when IP was done with another C-terminus directed anti-Nanog Ab, i.e., the R&D goat pAb, followed by WB using eBioscience mAb, we again detected the 42 kD Nanog protein in NTERA-2 NE (Fig. 3D, lane 3). Finally, IP with the eBioscience mAb followed by WB with the R&D goat pAb also detected the 42 kD Nanog protein (not shown). In ALL these IP experiments, the IP products showed an enrichment compared to WB using NTERA-2 NE (Fig. 3A–D; Fig. S2A–B). Since in all these experiments, IP was performed with one anti-Nanog Ab whereas WB with another, *the results provide further evidence that the major Nanog protein species in NTERA-2 cells migrates at* ∼*42* *kD on WB analysis* (see below for discussion on IP results in somatic cancer cells). Note that other than the 42 kD protein band, the IP did not pull down appreciable amount of 35 kD or other high M.W bands (Fig. 3A–D), likely due to much lower amounts of these protein species.

Subsequently, we performed IP and MS ID experiments in which we prepared **2** **mg** of NTERA-2 NE and immunoprecipitated the Nanog proteins using the R&D goat pAb, which pulled down three bands whose corresponding molecular masses were approximately 100 kD, 75 kD, and 55 kD, respectively (Fig. 3E). These three gel bands, together with the gel area corresponding to 42 kD (surprisingly, the 42 kD major band routinely detected on WB was not the major band pulled down by the R&D goat pAb), which were labeled as NTRD1 to NTRD4 (Fig. 3E), were cut out, and the proteins eluted and identified by LC-MS/MS (see Methods). We recovered Nanog peptides from all four gel slices, among which the NTRD4, corresponding to 42 kD region, showed the highest peptide spectral counts (Fig. 3F; Fig. S2C). Most recovered peptides were mapped to the N-terminus and homeodomain of the Nanog protein (Fig. S1C) although the IP Ab used was the R & D goat pAb directed against the C-terminus (Fig. 1A).

### Tandem IP and MS identify Nanog protein species of 20–70 kD in NTERA-2 cells

**Figure 4:**
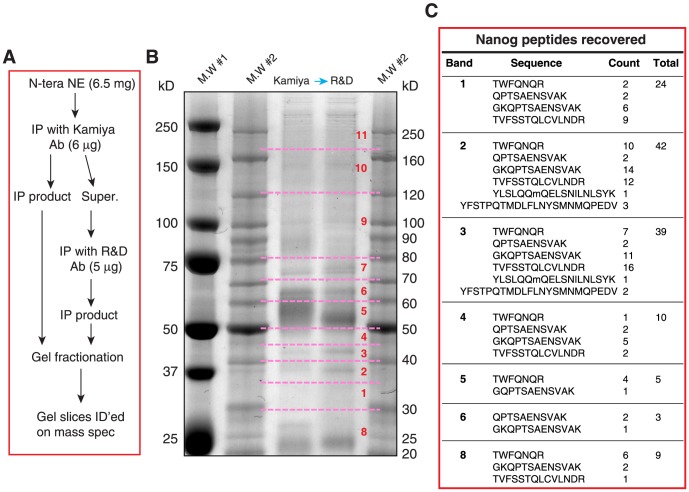
Mass spectrometry analysis of Nanog peptides recovered from NTERA-2 NE. (**A**) Flow chart of tandem IP and the sample preparation for LTQ mass spec analysis. (**B**) The gel image of SYPRO Ruby staining. The NE from NTERA-2 cells was used to perform the tandem IP with two anti-Nanog antibodies (i.e., Kamiya rabbit pAb and R&D goat pAb). The immunoprecipitates were separated by SDS-PAGE and the gel was stained with SYPRO Ruby. Gel slices as indicated (dashed lines and numbers) were cut out to elute proteins for LTQ mass spectrometry analysis. M.W#1 and M.W#2 were two protein markers. (**C**) Nanog peptides (sequences and total counts indicated) recovered for each gel slice.

*In the second set of protein ID experiments*, we employed tandem IP coupled with MS to identify the Nanog protein species in NTERA-2 cells (Fig. 4). We employed **6.5** **mg** NTERA-2 cell NE in tandem IP with two anti-Nanog antibodies, i.e., the Kamiya Rb pAb followed by R&D goat pAb (Fig. 4A). Eleven gel slices covering from ∼20 kD all the way to >250 kD were cut out and proteins eluted and subjected to LTQ MS analysis (Fig. 4B). We recovered Nanog peptides in gel slices 1, 2, 3, 4, 5, 6, and 8 whose M.W ranged from ∼20 kD to 70 kD. Most recovered peptides were also mapped to the N-terminus and homeodomain (Fig. S1D). The highest spectral counts of Nanog peptides were recovered from gel slices 2 and 3, corresponding to 35–45 kD (Fig. 4B–C). These results indicate that *in NTERA-2 cells, Nanog protein migrates at apparent molecular masses of 20*–*70* *kD*. Since the numbers of Nanog peptides recovered from gel slices 2 and 3 are very similar but on WB all anti-Nanog Abs preferentially detects the ∼42 kD band (in gel slice 3), these results further suggest that: 1) *the endogenous Nanog protein species migrate at various molecular masses probably by adopting distinct protein conformations and modifications (such as post-translational modifications or PTM, cleavage, etc), which are recognized by different anti-Nanog Abs*; and *2) the conformation of the 42* *kD Nanog is preferentially recognized by most anti-Nanog antibodies*.

### Exogenous NanogP8 protein is recognized by the 8 anti-Nanog1 Abs and migrates at 42 kD on WB

The above WB, siRNA knockdown, and two sets of MS–based protein ID experiments indicate that the Nanog1 protein in NTERA-2 cells migrates, on reducing SDS-PAGE, at multiple apparent molecular masses (most abundant at 35–45 kD) with the 42 kD band being the predominant band on WB. Next, we turned our attention to Nanog protein(s) in somatic cancer cells. There exists strong experimental evidence [15], [17], [31], [32], [34], [46], [63] that somatic cancer cells preferentially express the retrogene *NanogP8* located on Chr. 15q14 (gi 47777342) (Fig. S1B). *NanogP8* is very similar to *Nanog1* at the mRNA level with only six reported nucleotide (nt) differences [14], [15]. Indeed, our own sequencing [15] and differential qRT-PCR [34] analysis has demonstrated that the *Nanog* mRNA in multiple somatic cancer cell types (including primary prostate tumors) is derived from the *NanogP8* locus.

**Figure 5:**
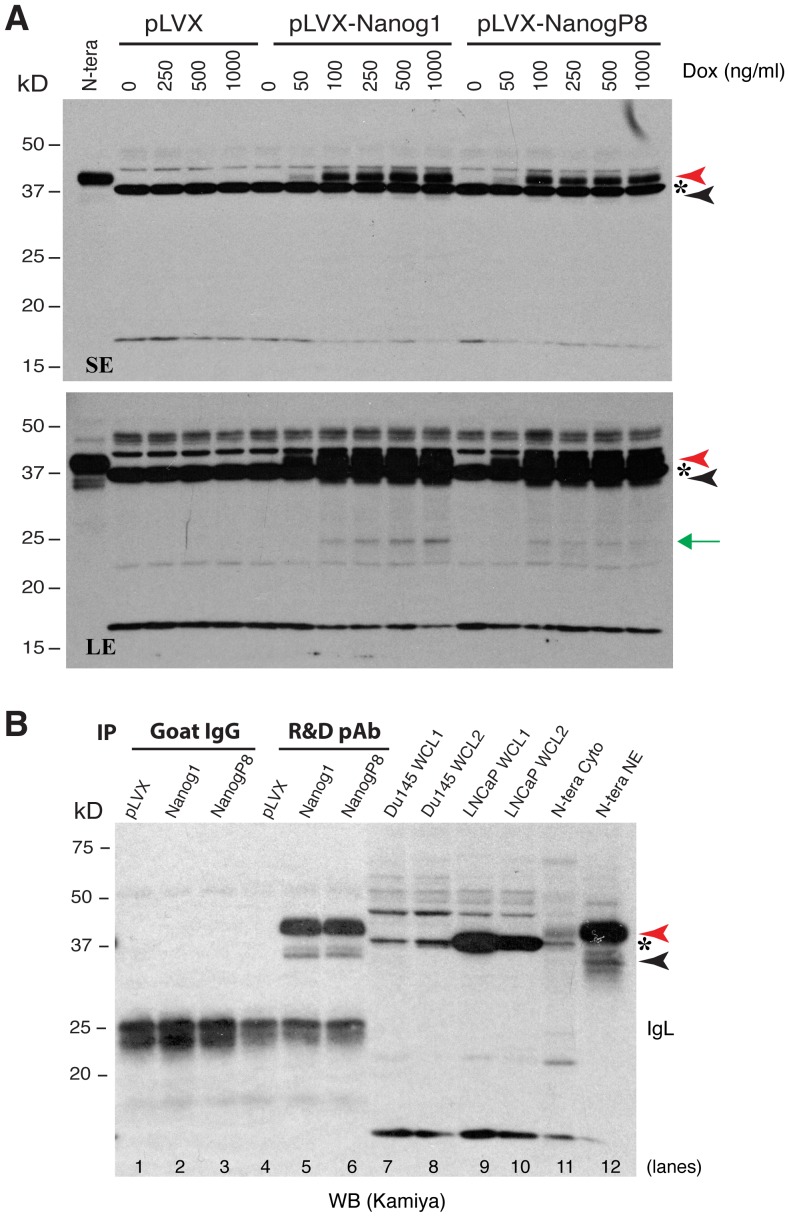
Exogenous NanogP8 expressed in LNCaP cells migrates mainly at 42 kD. (**A**) Exogenous NanogP8 migrates at 42 kD on WB. Whole cell lysate (80 µg/lane) prepared from control LNCaP (pLVX) or Nanog1/NanogP8 overexpressing LNCaP cells [34] in the presence of increasing amounts of doxycycline (Dox) was used in WB with the Kamiya anti-Nanog rabbit pAb. The red and black arrowheads indicate the main 42 kD and minor 35 kD Nanog bands, respectively. Green arrow, a ∼28 kD band that also increased upon Dox induction. Asterisk, a non-specific band. N-tera, NE of NTERA-2 cell; SE, short exposure; LE, long exposure. (**B**) The exogenous 42 kD and 35 kD bands could be immunoprecipitated down by the R&D anti-Nanog pAb. Whole-cell lysate (WCL; 500 µg) derived from pLVX, pLVX-NANOG1 and pLVX-NANOGP8 LNCaP cells [34] were used in IP with the R&D anti-Nanog goat pAb (goat IgG used as the control) followed by WB with Kamiya anti-Nanog rabbit pAb. The red and black arrowheads indicate the main 42 kD and minor 35 kD Nanog bands, respectively. Asterisk, a non-specific band. NE, nuclear extract; cyto, cytosol protein. Note that the 42-kD and the 35-kD protein bands only from pLVX-NANOG1 and pLVX-NANOGP8 LNCaP cells (but not from LNCaP-pLVX cells) were IP'ed down and detected on WB (lanes 5,6). Goat IgG did not pull down any specific bands. Also, WCL from two batches (1 and 2) of Du145 s and LNCaP cells (80 µg/lane) did not reveal the 42 kD and 35 kD protein bands on WB (lanes 7-10). The 37 kD non-specific band (asterisk) was not immunoprecipitated down by the R&D goat pAb (lane 5–6).

The *NanogP8* mRNA is predicted to encode a protein ∼99% identical to the Nanog1 protein in ES and EC cells except for one aa difference, i.e., **Q253H** [15]. Our earlier sequencing studies also identified, in somatic cancer cells, 3 nt changes or polymorphisms that could potentially lead to aa changes, i.e., **L61P**, **D64Y**, and **N130S** [15]. *Very little is known about the biochemical properties of NanogP8 protein in cancer cells*. To address this important deficiency, we first asked whether NanogP8 protein could be recognized by the 7 anti-Nanog1 Abs (except the BioLegend Ab) by taking advantage of our recent K14-NanogP8 transgenic (Tg) animal model in which the NanogP8 cDNA derived from a primary PCa (i.e., HPCa5) was driven by a cytokeratin 14 promoter [64]. The IHC staining revealed that all 7 anti-Nanog1 Abs detected NanogP8 in the nuclei of Tg keratinocytes (Fig. S3). Of interest, the NanogP8 protein in the Tg tissues migrates, on denaturing SDS-PAGE and WB, at 42 kD [64]. Strikingly, both NTERA-2 *Nanog1* and HPCa5 *NanogP8* cDNAs, when overexpressed from in LNCaP PCa cells using a doxycycline inducible system [34], encoded proteins that also migrated at 42 kD on WB using the Kamiya pAb (Fig. 5A, red arrowheads), just like the endogenous Nanog1 protein in NTERA-2 cells (Fig. 5A). These results were consistent with our earlier findings [34]. On longer exposure of the films, the 35 kD band (Fig. 5A, bottom panel, black arrowhead) and a 28 kD band (Fig. 5A, bottom panel, green arrow) could also be detected. Note that the Kamiya pAb detected a prominent non-specific ∼38 kD protein band (Fig. 5A, asterisks), which was identified by mass spec as the sorbital dehydrogenase (data not shown). Both 42 kD and 35 kD proteins could be immunoprecipitated down by the R&D goal pAb and detected on WB by the Kamiya pAb (Fig. 5B, lanes 5 and 6), which co-migrated with the endogenous Nanog1 proteins in NTERA2 NE (Fig. 5B, lane 12; see below for further discussions on NanogP8 in cancer cells).

### Biochemical characterizations of recombinant Nanog1 (rNanog) and NanogP8 (rNanogP8) proteins using the 8 anti-Nanog Abs

**Figure 6:**
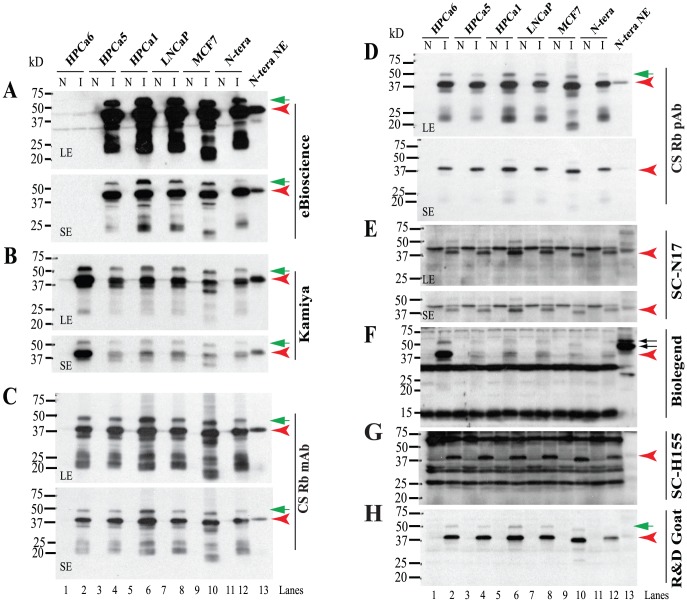
Reactivity of rNanogP8 and rNanog proteins towards 8 anti-Nanog Abs. WB analysis using 8 anti-Nanog Abs (A-H). Cell types from which the initial cDNAs were cloned are indicated on top. Individual Abs are indicated on the right and M.W on the left. For some Abs, both a long (LE) and short (SE) exposures were shown. N: non-induced; I: induced by IPTG (see Methods). The red arrowheads in each panel indicate the 42 kD major Nanog protein and green arrows point to minor upper bands. In panel F, the two arrows point to the ∼48/54 kD doublets recognized by the BioLegend Rb pAb.

Next, we freshly made rNanogP8 proteins from the cDNAs of a cultured PCa cell line (LNCaP), three primary prostate tumors (HPCa1, 5, and 6), and one breast cancer cell line (MCF7) (Fig. 6). For comparison, we also made rNanog1 from NTERA-2 cells (Fig. 6). The rNanogP8 and rNanog1 proteins were run on WB and probed with the 8 anti-Nanog Abs, which again exhibited distinct reactivity (Fig. 6). We observed that the majority of rNanogP8 proteins behaved overall similarly to the rNanog1 protein. Like in NTERA-2 NE, most Abs detected a major 42 kD band (Fig. 6, red arrowheads) as well as a minor upper band of either ∼48 kD (SC N17, R&D goat pAb, CS Rb pAb and Rb mAb) or ∼55 kD (Kamiya, BioLegend and eBioscience) (Fig. 6, green arrows). Five Abs (eBioscience, Kamiya, CS pAb and mAb, and R&D pAb) showed clean reactions on WB whereas the other three antibodies (BioLegend pAb, SC H-155, and SC N17) detected many non-specific bands (i.e., bands detected under non-induced conditions) (Fig. 6).

Also, different rNanog proteins displayed differential reactivity to different anti-Nanog Abs. Five antibodies (CS Rb pAb and Rb mAb, SC N17, SC pAb H-155, and R&D goat pAb) detected all six rNanog proteins with similar efficiency but two antibodies (Kamiya pAb and BioLegend pAb) preferentially reacted with the HPCa6 rNanogP8 whereas the eBioscience mAb, remarkably, did not recognize the HPCa6 rNanogP8 at all (Fig. 6A). Very interestingly, the MCF7 rNanogP8 consistently migrated slightly faster, at ∼37 kD, than other rNanogP8 proteins (Fig. 6). Taken together, these results suggest that **1** ) *the rNanogP8 proteins, like endogenous Nanog1 and rNanog1 in NTERA-2 cells, seem to be able to adopt different conformations that exhibit varying mobility on reducing SDS-PAGE*; **2** ) *somatic cancer cells may encode different ‘isoforms’ of NanogP8 due to sequence polymorphisms (such as those in HPCa6 and MCF7 cells)*; *and* **3** ) *NanogP8 proteins of different conformations or polymorphisms may be differentially recognized by various anti-Nanog Abs with the 42 kD still being the major band on WB*.

### rNanogP8 proteins also migrate at multiple M.W species as revealed by IP followed by MALDI-TOF/TOF

**Figure 7:**
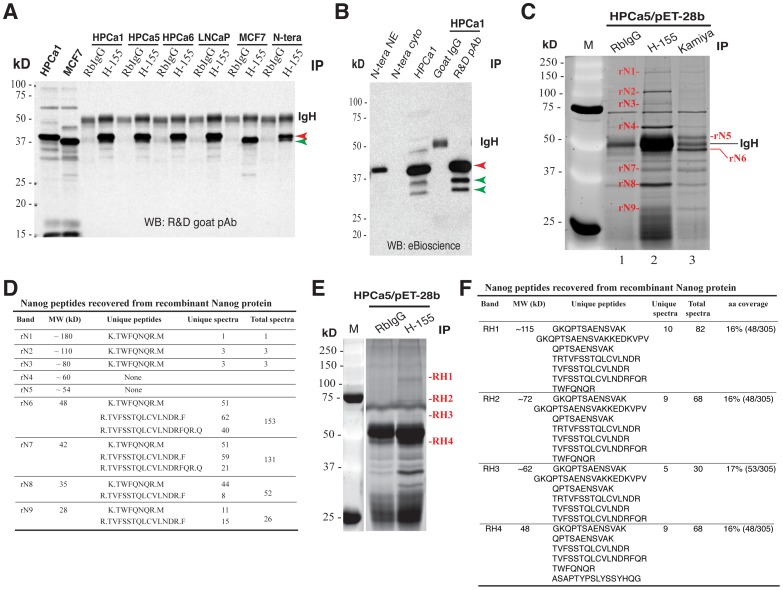
rNanogP8 protein ID using IP and mass spectrometry. (**A**) IP in rNanogP8 proteins using the SC pAb H-155 followed by WB using the R& D goat pAb. RbIgG was used as the control Ab. Red arrowhead, the 42 kD band; green arrowhead, the faster migrating major band in MCF7 rNanogP8; IgH, IgG heavy chain. (**B**) IP using the R&D goat pAb followed by WB using the eBioscience mAb. Goat IgG was used as the control Ab. Red arrowhead, the 42 kD band; green arrowheads, the two lower bands; IgH, IgG heavy chain. In this experiment, NTERA-2 NE and cytosol and HPCa rNanogP8 were also loaded in WB analysis. (**C–D**) Mass spectrometry ID of rNanogP8 proteins. The HPCa5 rNanogP8 protein made in pET-28b was used in IP with either H-155 or Kamiya Rb pAb (RbIgG as the Ab control). The immunoprecipitates were subjected to SDS-PAGE, stained with SYPRO Ruby, and 9 bands (rN1 - rN9) cut out for protein ID (C). M, protein marker. The identified peptides were presented in D. (**E–F**) Mass spectrometry ID of rNanogP8 proteins in a separate experiment using conditions as above. Four bands (RH1–RH4) were cut out for protein ID (E) and identified peptides presented in F.

**Figure 8:**
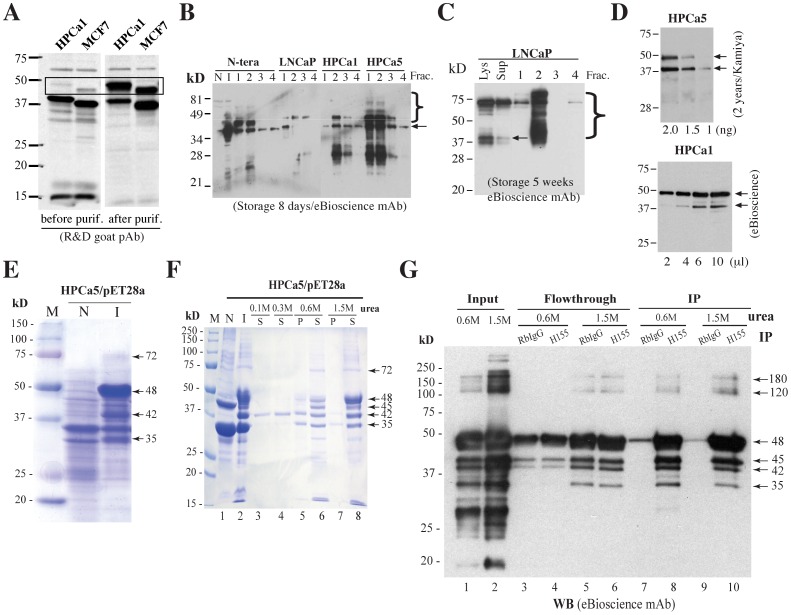
Evidence that rNanogP8 proteins can spontaneously form high M.W species. (**A**) rNanogP8 proteins from HPCa1 and MCF7, before and after purification, were used in WB using the R&D goat pAb. Note that prior to purification, the HPCa1 and MCF7 rNanogP8 proteins migrated at ∼42 kD and ∼37 kD, respectively, with a minor upper band detected for both proteins. After purification, the intensity of the upper bands (in the rectangle) became significantly stronger. (**B–C**) rNanogP8 proteins (from the indicated cell types) stored at −80°C for 8 days (B) or 5 weeks (C) were used in protein purification. Aliquots (20 µl) of 4 fractions (Frac.) for each sample, together with NTERA-2 non-induced (N) or induced (I) bacterial lysate (B) or LNCaP total bacterial lysate (Lys) or supernatant (Sup) (C), were used in WB with the eBioscience mAb. The arrows indicate the ∼42 kD rNanogP8 proteins and right-hand brackets indicate high M.W ladders. (**D**) The HPCa5 and HPCa1 rNanogP8 proteins stored for 2 years was utilized in WB with the Kamiya pAb and eBioscience mAb, respectively. (**E**) Coomassie brilliant blue R-250 staining of rNanogP8 protein made from HPCa5 cDNA in pET-28a. The arrows indicate the high levels of rNanogP8 proteins under the induced conditions. M, protein marker; N, non-induced; I, induced by IPTG. (**F**) Coomassie brilliant blue R-250 staining of HPCa5 rNanogP8 proteins from refolding dialysis experiment. The inclusion bodies of rNanogP8 protein were first dissolved in 7 M urea and then subjected to dialysis against decreasing concentrations of urea. N, non-induced; I, induced by IPTG; S, soluble portion; P, pellet (precipitated portion). Arrows indicate rNanogP8 proteins of different molecular weights. (**G**) IP analysis confirms that the refolded bands are rNanogP8. The dialysis samples containing 1.5 M or 0.6 M urea were subjected to IP with the SC pAb (H-155) or RbIgG (control) followed by WB analysis with the eBioscience mAb. Arrows indicate rNanogP8 proteins of different molecular weights.

We performed IP experiments in rNanog1 protein from NTERA-2 and rNanogP8 proteins from HPCa1, HPCa5, HPCa6, LNCaP and MCF7 (Fig. 7A–C; data not shown). IP with the SC Rb pAb H-155 pulled down the major 42 kD (Fig. 8A, red arrowhead; green arrowhead pointed to the faster migrating major band in MCF7 cells) and some minor bands (apparent upon longer exposure). IP with the R&D goat pAb also pulled down the major 42 kD (Fig. 7B, red arrowhead) and two lower bands (Fig. 7B; green arrowheads). Similarly, IP with the Kamiya pAb and eBioscience mAb pulled down rNanogP8 proteins (Fig. 7C; data not shown). These results indicate that like the endogenous Nanog1 protein in NTERA-2 NE, rNanogP8 proteins derived from cDNAs in somatic cancer cells can be readily immunoprecipitated down by various anti-Nanog Abs and that the 42 kD protein also represents the major protein band on WB.

Subsequently, we immunoprecipitated the HPCa5 rNanogP8 using two anti-Nanog antibodies, i.e., SC pAb H-155 and Kamiya pAb, and, after SDS-PAGE, we stained the gel with SYPRO Ruby (Fig. 7C). Compared with rabbit IgG control, at least 9 specific bands from ∼28 kD to ∼180 kD were identified by the two anti-Nanog Abs (Fig. 7C). Mass spectrometry ID experiments revealed that 7 of the 9 bands (named rN1-rN9) with M.W of approximately 28 kD, 35 kD, 42 kD, 48 kD, 80 kD, 110 kD, and 180 kD, contained Nanog peptides (Fig. 7D). A repeat IP and MALDI-TOF/TOF experiment similarly uncovered Nanog peptides at approximately 48, 62, 72, and 115 kD (Fig. 7E-F).

### Evidence that rNanogP8 proteins spontaneously form high M.W species

During multiple experiments with rNanogP8 proteins, we frequently observed <40 kD protein bands, which might have resulted from degradation. However, we also consistently observed >40-kD Nanog protein bands (Fig. 5; Fig. 6B; Fig. 7C–F), particularly during protein purification with stored samples (Fig. 8A–D). For instance, the HPCa1 rNanogP8, prior to purification, migrated at ∼42 kD with a minor band at 48 kD (Fig. 8A). Strikingly, after purification (i.e., with increased protein concentration), the 48 kD band became predominant (Fig. 8A). Likewise, the MCF7 rNanogP8 migrated at ∼37 kD with a minor band at ∼45 kD, the latter of which significantly increased upon purification (Fig. 8A). These spontaneously formed high M.W rNanogP8 species were even more apparent when the samples stored at −80°C for different time intervals were used in protein purification (Fig. 8B–C). For example, distinct tapering ladders of ≥40-kD were observed in the purified NTERA-2, LNCaP, HPCa1, and HPCa5 rNanog(P8) proteins stored for 8 days (Fig. 8B). In LNCaP rNanogP8 stored for 5 weeks (prior to being used in purification), it appeared that most 42 kD and intermediate protein species had migrated to ∼72 kD (Fig. 8C). When purified rNanogP8 stored long-term (e.g., 2 years) were used in WB, we routinely detected two bands at 42 kD and 48 kD with some minor bands in between (Fig. 8D).

When we subcloned the full-length HPCa5 NanogP8 cDNA into the pET-28a bacterial expression vector that allows high levels of recombinant protein expression, we detected at least 4 obvious recombinant rNanogP8 bands at approximately 75 kD, 48 kD, 42 kD and 35 kD upon IPTG induction, among which the 48 kD band had the highest yield (Fig. 8E). We found that the recombinant HPCa5 rNanogP8 protein was mostly present in the inclusion bodies. Consequently, we conducted a ‘refolding’ analysis in buffers containing various concentrations of urea. After isolating the inclusion bodies of the HPCa5 rNanogP8 protein from pET-28a *E. coli*, we first used 7 M urea solution to dissolve the inclusion bodies, and then carried out the refolding dialysis experiments, in decreasing concentrations of urea (i.e., 1.5 M, 0.6 M,0.3 M and 0.1 M), to let the denatured rNanogP8 protein refold into its potentially native form (Fig. 8F). We next conducted SDS-PAGE with these dialysis samples and stained with Coomassie brilliant blue R-250. As shown in Fig. 8F, there were at least 5 soluble rNanogP8 bands with the M.W of approximately 72 kD, 48 kD, 45 kD, 42 kD and 35 kD in the 1.5 M urea dialysis buffer and the 48 kD band was the most prominent (Fig. 8F, lane 8). In 0.6 M urea, the abundance of the 48 kD band was dramatically reduced (Fig. 8F, lane 6). Strikingly, the 42 kD HPCa5 rNanogP8 protein remained soluble even in 0.3 M and 0.1 M urea dialysis buffers (Fig. 8F, lane 3 and 4), suggesting that *the 42* *kD band might represent the ‘native’ (i.e., most soluble) NanogP8 protein*. Finally, we immunoprecipitated the 0.6 M and 1.5 M urea dialysis samples using the SC Rb pAb H-155 and analyzed them on WB. The H-155 Ab immunoprecipitated down 6 bands with M.W ranging from 35 kD to ∼180 kD (Fig. 8G, lanes 8 and 10).

### Anti-Nanog1 antibodies fail to IP down endogenous NanogP8 proteins in long-term cultured or xenograft-derived somatic cancer cells

Many studies have reported Nanog protein expression in somatic cancer cells (see Introduction; Table 1) [63], Given that most somatic cancer cells predominantly or exclusively express *NanogP8* mRNA, it stands to reason that any putative Nanog protein they express should be NanogP8 protein. As frequently only a cropped strip of WB image or a ‘representative’ IHC panel is shown in many of these studies, it is unclear whether the Nanog protein reported on WB or IHC truly represents NanogP8. In preliminary studies, we carried out IP experiments using 4 anti-Nanog Abs, i.e., Kamiya Rb pAb, CS Rb mAb, SC H-155 Rb pAb, and R&D goat pAb using ∼500 µg NE prepared from several cultured cancer cell lines including PCa (Du145), breast cancer (MCF7), colon cancer (Colo320), and melanoma (WM562) (Fig. 3A–D; Fig. S2A). Although these Abs readily immunoprecipitated down the 42 kD endogenous Nanog protein in NTERA-2 NE (Fig. 3A–D; Fig. S2), and the rNanogP8 proteins made from somatic cancer cells (Fig. 7), they did not pull down the 42 kD or other NanogP8 proteins in cultured cancer cell NE (Fig. 3A–D; Fig. S2A). Similarly, the Kamiya pAb did not immunoprecipitate down NanogP8 in the NE of LAPC4 and LAPC9 PCa xenografts (Fig. 3A). Finally, although the R&D pAb immunoprecipitated down both 42 kD and 35 kD Nanog1 and NanogP8 proteins in Dox-induced LNCaP cells, it did not pull down any of these proteins in un-induced cells (Fig. 5B). In fact, regular WB using cytosol and NE from these cancer cells did not reveal the major 42 kD Nanog, which was always readily detected in NTERA-2 NE (Fig. 3A–D; Fig. S2A; Fig. 5). It should be noted that the Kamiya pAb recognized a non-specific ∼38 kD band, which was not NanogP8 as it was not pulled down by the R&D goat pAb (Fig. 5B).

## Discussion

The current project was undertaken to characterize the biochemical properties of Nanog protein in EC and, potentially, in somatic cancer cells. We have made the following novel findings. ***FIRST***, Nanog1 in NTERA-2 cells is detected on WB primarily as a 42 kD protein band by 7 of the 8 Abs tested. ***SECOND***, Nanog1 in NTERA-2 cells exists at multiple M.W species at up to ∼100 kD (Table 2). Some of these Nanog1 species (e.g., the 35 kD) appear to be as abundant as the 42 kD protein (based on mass spec) although most of them are less abundant. ***THIRD***, the 42 kD Nanog1 band in NTERA-2 cells can be reliably and readily immunoprecipitated down by most Abs and subsequently detected on WB. ***FOURTH***, most anti-Nanog1 Abs tested recognize the EXOGENOUS NanogP8 protein in Tg mouse tissues and human cancer cells and the exogenous NanogP8 in these settings also migrates at 42 kD. ***FIFTH***, both rNanog1 and rNanogP8 proteins are detected, on WB, mainly as the 42 kD band. ***SIXTH***, like endogenous Nanog1 protein in NTERA-2 cells, rNanog1/rNanogP8 proteins can also be detected as multiple M.W species at up to ∼180 kD. ***SEVENTH***, rNanogP8 appears to be able to spontaneously form high M.W protein species. ***EIGHTH***, the epitope of the eBioscience mAb raised against the full-length human Nanog1 protein is mapped to the L61 region in the N-terminus. ***FINALLY***, anti-Nanog1 antibodies fail to IP down the endogenous 42 kD (or 35 kD or other) NanogP8 proteins in long-term cultured or xenograft-derived somatic cancer cells.

**Table 2:** Nanog proteins in NTERA-2 human EC cells.*

Ab	IP/MALDI	Tandem IP/LTQ	IP	WB	Knockdown
	(Fig. 3E-F)	(Fig. 4)	(Fig. 3A-D; Fig. S2)	(Fig. 1)	(Fig.2)
**eBioscience mAb**				42 kD>>35 kD>>65 kD	
**Kamiya Rb pAb**		∼25–70 kD	42 kD	42 kD>>35 kD; 48, 65, 100 kD	42 kD>>35 kD; 48, 65, 75, 90 kD
**CS Rb pAb**				42 kD>>35 kD>>65 kD	42 kD>>35 kD; 65 kD
**CS Rb mAb**			42 kD	42 kD>>35 kD; 28,32, 70, 100 kD	
**SC N-17 Rb pAb**				42 kD>>35 kD>55/58 kD	
**Biolegend Rb pAb**				48 kD>35 kD>55 kD	
**SC H-155**			42 kD	42 kD>>35 kD; 55 kD, 58 kD	
**R&D goat pAb**	42, 60, 75, 100 kD	∼25–70 kD	42 kD	42 kD>>35 kD; 28, 65, 70 kD	

*Presented are the results of various experiments using the 8 anti-Nanog Abs. The Biolegend Ab is the only Ab that does not recognize the 42 kD band as the major protein band and does not recognize the 35 kD band. Presented molecular masses are all estimated based on their migrations on SDS-PAGE.

Nanog protein has 305 aa with a predicted M.W of ∼35 kD. WB using NE, siRNA-mediated knockdown, and IP followed by MS-based peptide ID, combined, provide UNEQUIVOCAL evidence that *the endogenous Nanog protein in NTERA-2 EC cells exists as multiple M.W species, migrating at <30* *kD (e.g., 28* *kD) all the way to* ∼*100* *kD* (Fig. 1–4; Table 2). WB analysis of NTERA-2 NE demonstrates that 7 of the 8 anti-Nanog Abs, raised against the N- or C-terminus of human Nanog protein, preferentially reacts with the major 42 kD and minor 35 kD Nanog1 proteins. These results are consistent with the two MS-based ID experiments showing that the gel slices of 35–45 kD contain the highest Nanog peptide counts (Fig. 3F; Fig. 4B–C), suggesting that the endogenous Nanog1 proteins in NTERA-2 cells are most abundant at 35–45 kD range. On the other hand, the 42 kD Nanog seems to be the preferred protein species recognized by all anti-Nanog Abs in both WB and IP, which raises the possibility that different Nanog protein species may adopt different conformations, which dictate their gel mobility as well as Ab reactivity. In support, we find that in addition to the 42 kD and 35 kD bands, various anti-Nanog Abs also recognize additional protein bands but with slightly different patterns (Table 2). Surely, although not all protein bands detected on WB in NTERA-2 NE are Nanog1 proteins, knockdown experiments together with MS-based protein ID do suggest that many of these bands represent authentic Nanog1 protein.

How could a predicted 35 kD nuclear protein migrate at as large as ∼100 kD on reducing and denaturing SDS-PAGE? *First of all*, the *Nanog1* gene has been reported to undergo alternative splicing, which could potentially generate Nanog proteins of different molecular masses [52], [57]. But alternative splicing mainly gives rises to smaller Nanog protein variants [57]. Furthermore, cDNA-derived Nanog1 and rNanog1 proteins also migrate at 42 kD or above, arguing against alternative splicing being a mechanism generating high M.W Nanog protein species. *Secondly*, Nanog protein in ES cells has been reported to function as homodimers formed through the interactions between the Nanog's WR domain, which could weigh ∼70–80 kD [12], [13]. Moreover, Nanog protein in ES cells exists in gigantic protein complexes [53]. These observations, however, do not seem to be able to explain our present results because Nanog homodimers and protein complexes would have been disrupted by the reducing agents and denaturant SDS. *Thirdly*, Nanog protein can undergo two types of post-translational modifications (PTM) including phosphorylation [39], [65] and ubiquitylation [66]. Indeed, in some of our analysis of endogenous Nanog1 in NTERA-2 cells, we frequently observed one or several bands right above the minor 35 kD Nanog protein (e.g., Fig. 1E and H; Fig. 3C; Fig. 5B), which likely represent the phosphorylated Nanog1 proteins. However, we believe that phosphorylation will unlikely explain dramatic M.W shifts of the Nanog1 protein (all the way up to ∼100 kD). More important, the rNanog protein made from the NTERA-2 cDNA in bacteria, which lack the above-mentioned PTM machinery, also primarily migrates at 42 kD and higher, thus arguing against PTMs as the major mechanisms.

Comprehensive studies using rNanog1 from NTERA-2 and rNanogP8 proteins from multiple somatic cancer cells further suggest that Nanog1 and NanogP8, which are >99% identical at the aa levels, seem to possess unique INTRINSIC biochemical properties that can allow them to adopt multiple protein conformations and consequently migrate at multiple apparent M.W. The rNanogP8 proteins made from 4 PCa (LNCaP, and HPCa1, 5, and 6) cell types all migrate at 42 kD as the major band on both WB (Fig. 6) and IP (Fig. 7A–B) analyses. Strikingly, IP of the rNanogP8 proteins using both an N-terminus directed Ab (i.e., Kamiya) and a C-terminus directed Ab (i.e., SC H-155) followed by MS have uncovered Nanog peptides from 28 kD all the way to ∼180 kD (Fig. 7C–F). Surprisingly, although the 42 kD protein represents the major band on WB, the highest number of Nanog peptides is recovered for the 48 kD band, which is recognized preferentially by the Kamiya Ab (Fig. 7C–D). In contrast, the SC H-155 Ab preferentially recognizes the 42 kD (the second highest peptide count) and 35 kD (the third highest peptide count) rNanogP8 proteins (Fig. 7C–D).

How could rNanogP8 proteins made in bacteria, just like endogenous Nanog proteins in NTERA-2 cells, migrate at multiple M.W species? Since the above-discussed experiment (Fig. 7C–F) is performed using two different Abs on the rNanogP8 proteins made from a single cancer cell type, i.e., HPCa5, the results strongly argue that the rNanogP8 protein, by itself, can adopt different conformations that are differentially recognized by different anti-Nanog Abs. In support, we have observed that the rNanog1 in NTERA-2 cells as well as the rNanogP8 proteins from somatic cancer cells can spontaneously form high M.W protein species during protein purification when the (local) concentrations of recombinant proteins dramatically increase (Fig. 8A–C). In further support, when rNanogP8 proteins are made in a bacteria strain that allows high levels of protein production, an apparent ladder of rNanogP8 protein species ranging from ∼35 kD to 180 kD is observed (Fig. 8E–G). In such experiments, the 48 kD protein represents the major NanogP8 species (Fig. 8E–F), which can be efficiently pulled down by the SC H-155 Ab (Fig. 8G). However, in the urea-assisted ‘denaturation and refolding’ analysis, the 42 kD rNanogP8 is the only protein species observed in the lowest urea-containing buffers whereas all other rNanogP8 species can only be seen in buffers containing >0.3 M urea (Fig. 8F), suggesting that *the 42* *kD rNanogP8 represents the ‘native’ and most stable (and most soluble) Nanog protein conformer*, explaining its preferential detection on WB in both NTERA-2 NE (Fig. 1) and rNanog1/rNanogP8 proteins (Fig. 6). This suggestion is consistent with the fact that even when Nanog1/NanogP8 cDNAs are overexpressed in cancer cells, the major protein detected on WB is 42 kD (Fig. 5). The intrinsic properties of Nanog1/NanogP8 proteins, to a certain degree, resemble c-Myc oncoprotein, which can be detected on WB at M.W from ∼40 kD to >60 kD [67], [68]. Precisely how the high M.W Nanog proteins are formed is currently being investigated in the lab.

The Nanog protein (i.e., NanogP8) has been reported to be overexpressed in tumors [18]–[27], [54]–[57], [63]. However, our preliminary IP experiments using NE extracts prepared from long-term cultured cancer cell lines or xenograft tumors fail to pull down the dominant 42 kD (or other) NanogP8 protein although all 5 anti-Nanog Abs can readily immunoprecipitate the 42 kD Nanog protein in the same amount (500 µg) of NTERA-2 NE (Fig. 3A-D; Fig. S2A; Fig. 5B). In fact, WB using NE or cytosol from these somatic cancer cells did not identify the 42 kD NanogP8 protein (e.g., Fig. 3A-D; Fig. 5). Several potential explanations may underlie this negative result. ***First***, long-term cultured or xenograft-derived somatic cancer cells express too low levels of NanogP8 to be detected by conventional IP with relatively small amounts of NE. ***Second***, long-term cancer cell cultures and perhaps most long-term xenografts contain too few NanogP8-expressing cells. This possibility is supported by our earlier studies showing NanogP8 expression in only a very small percentage of cultured cancer cells [15] and primary prostate tumors and early-generation xenograft prostate tumors expressing NanogP8 mRNA at levels several orders of magnitude higher than in long-term cultured cancer cells [34]. ***Third***, endogenous NanogP8 protein in somatic cancer cells is different from the Nanog1 protein in NTERA-2 cells such that the NanogP8 is not recognized well on IP and WB by anti-Nanog1 Abs. ***Finally***, the endogenous NanogP8 protein in somatic cancer cells may undergo unique PTMs and consequently possess a very short half-life. All these (and other) possibilities are currently been pursued in our lab.

## Supporting Information

Figure S1 **Genomic organization of** ***Nanog*** **and** ***NanogP8*** **genes and Nanog peptides recovered from mass spectrometry analysis in N-tera NE.** (**A**) Schematic of *Nanog1* gene. Chr, chromosome; E, exon; TSS, transcription start site; UTR, untranslated region. The 22-bp region unique to the 3′-UTR of *Nanog1* (vertical black bar) was indicated, which was used in our earlier differential qRT-PCR analysis [34]. (**B**) Schematic of the *NanogP8* retrogene, which lacks introns and is located on Chr. 15q14. The red asterisk indicates the AlwN1 restriction site located on nt144. (**C–D**) Nanog protein structure. The 305 aa Nanog protein contains 5 protein domains, i.e., ND (N-terminus domain), HD (homeodomain), WR (tryptophan-rich domain), and CD1 and CD2 (C-terminus domain 1 and 2). Most Nanog peptides recovered in N-tera NE upon LC-MS/MS (Fig. 3E–F) and tandem IP/mass spectrometry (Fig. 4) are mapped to the ND and HD (indicated below; the numbers in the parentheses refer to the number of peptides).
(EPS)

Figure S2 **IP studies and Nanog protein ID in NTERA2 NE.** (**A**) N-tera NE was used in IP with the Kamiya pAb followed by WB with the R&D goat pAb. Lanes 1–7 were regular WB using cytosol (C) or NE from the cells indicated or using whole cell lysate (WCL) from NTERA2. The top and bottom panels are long exposure (LE) and short exposure (SE), respectively. Red arrowhead indicates the 42 kD Nanog and black arrowhead the 35 kD band (both circled) whereas green arrows indicate additional bands detected on WB only in NE. IgH, IgG heavy chain (∼53 kD). (**B**) NTERA2 NE was used in IP with the SC pAb (H-155) followed by WB with the eBioscience mAb. Red arrowhead, the 42 kD Nanog band; IgH, IgG heavy chain. (**C**) Representative mass spectra of peptides detected in the 4 gel slices labeled as NTRD1 – NTRD4.
(EPS)

Figure S3 **HPCa5-derived NanogP8 expressed in transgenic mouse epidermis is recognized by all 7 anti-Nanog Abs tested.** Immunohistochemistry of skin sections stained with 7 anti-Nanog antibodies. WT, wild-type; TG, K14-NanogP8 transgenic mouse [64]. Boxes areas were enlarged and shown in insets. Dark brown color indicates the positive cells; blue color indicates nuclear counterstaining.
(TIF)
