# Supplementary figures and images for: Nanog1 in NTERA-2 and Recombinant NanogP8 from Somatic Cancer Cells Adopt Multiple Protein Conformations and Migrate at Multiple M.W Species
Source: PLoS One. 2014 Mar 5;9(3):e90615. doi: 10.1371/journal.pone.0090615 (PMC3944193; doi:10.1371/journal.pone.0090615)

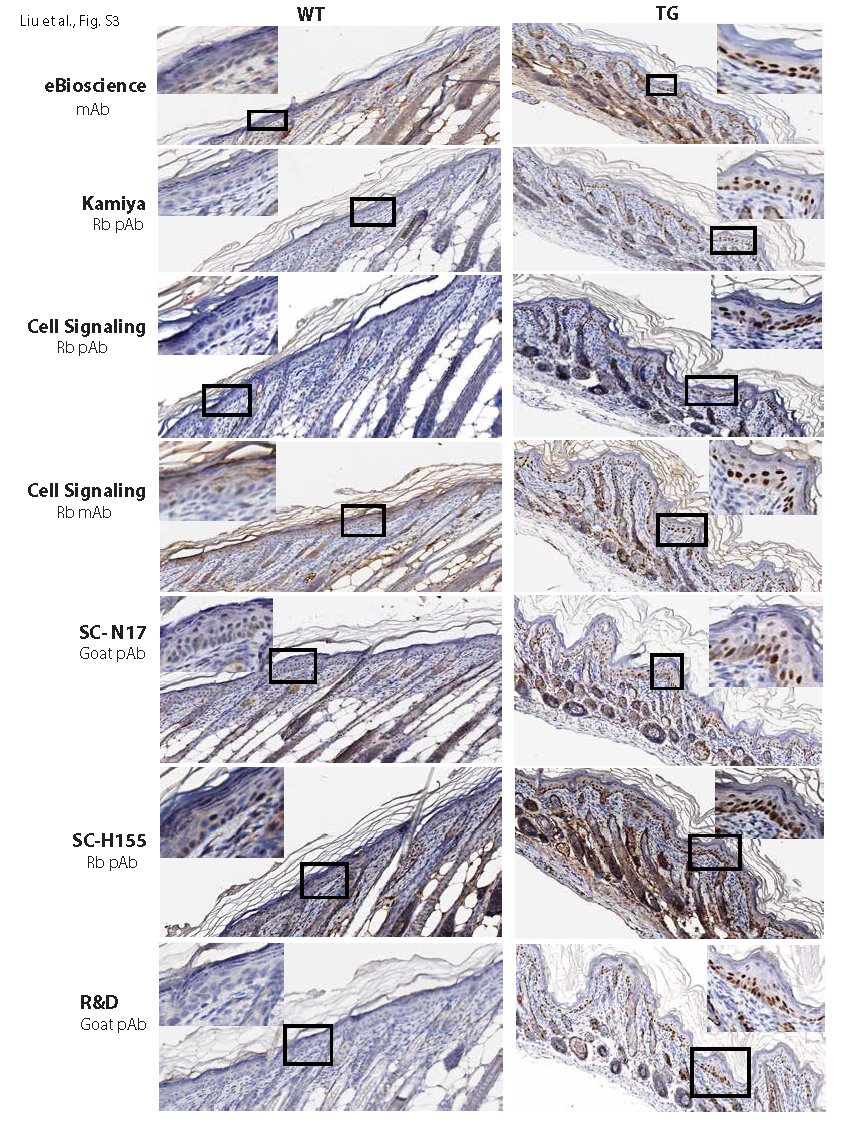

Supplement: Figure S3 — HPCa5-derived NanogP8 expressed in transgenic mouse epidermis is recognized by all 7 anti-Nanog Abs tested. Immunohistochemistry of skin sections stained with 7 anti-Nanog antibodies. WT, wild-type; TG, K14-NanogP8 transgenic mouse [64]. Boxes areas were enlarged and shown in insets. Dark brown color indicates the positive cells; blue color indicates nuclear counterstaining. (TIF) [file pone.0090615.s003.tif]
